# Provings*Kansas State Homœopathic Society, 1896.

**Published:** 1896-12

**Authors:** P. Dietrick

**Affiliations:** Kansas City, Kansas


### PROVINGS.*

P. DIETRICK, M. D., KANSAS CITY, KANSAS.

What are homœopathic provings? According to the law of simillimum the real effect of drugs upon the human system can be ascertained only by administering the drugs to healthy persons and observing the result. All drugs produce symptoms, and the totality of these symptoms constitutes the effect of the remedy.

Is it an easy matter to make homœopathic provings? Not at all. The work of proving is a difficult task, and the large majority of mankind is unfit to engage in this work.

Why? Because, in the first place, only healthy persons are available as provers, and this excludes more than seventy-five per cent. of our people. We speak of perfect health as if it were nothing unusual and could be found everywhere; but alas! this is not so, and a state of perfect health is an ideal existence, which is rarely found among civilized people.

A second difficulty is the fact that not all provers are able to give an accurate account and description of all the symptoms which may be produced upon them by the drug. The aid of chemistry and microscopy may be necessary to determine correctly whether the bodily secretions and excretions remain normal or whether they are abnormal, and in regard to the effects upon the eye, ear, nose, throat, and genital organs, a specialist only could describe accurately the changes which may take place in those parts. An inborn, natural faculty enables many persons to do certain kinds of work in a superior manner, and so I believe it was with Dr. Hahnemann. His

---

* Kansas State Homœopathic Society, 1896.

mind was of a peculiar, penetrating character, and his observations in provings and verifications are accurate and reliable records. Any one who doubts the facts of his records may demonstrate their truth by following his advice: "Macht's nach, aber macht's genau nach." His clear, penetrating mind was of the character of the Rœntgen X rays, for he saw and understood clearly the different phenomena of life, health, and disease, when all others around him groped in darkness, superstition, and error.

Many times we hear him praised as a great medical reformer, but I think the name reformer fails to do justice to him. He was more than a reformer, he was an originator, a founder, a corner-stone. Up to his time, and outside of his system, there never was any science in therapeutics, and surely he did more than reform that miserable theoretical guessing and empirical nonsense in medicine existing at his time. He was the founder of scientific medicine, and upon the principles of Homœopathy rests the whole progress in modern therapeutics. Never mind what new and formidable diseases may turn up to destroy our race, the homœopathic materia medica will point out clearly and unmistakably remedies that heal and cure the afflicted. Let us rejoice that we belong to those who know that Homœopathy is a true natural law, and that we remain to be at the top of scientific therapeutics, as long as we closely adhere to the principles which Hahnemann laid down in his *Organon*.

Verifications. What does that signify? It means, that in clinical medicine, at the bedside of patients, in hospitals and in office practice we must verify the truth of the maxim that remedies are curative according to the law of therapeutics. That is an easy matter, is it not? The large majority of homœopathic physicians have never made a proving, but verifications they make daily. However, some difficulties present themselves also here. Namely, how do you know that the patient got well from our prescription? Are not many diseases self-limited, and the patient gets well without any medicine at all, if we only take proper care of him? And does a change of habits, diet, climate, and environments not produce a cure without any medicine in many instances? And do not some patients get well in cases when, according to our knowledge, a wrong treatment has been adhered to all through the sickness, so that we could truthfully say, the patient got well in spite of the treatment?

All this is true, and, therefore, it is not an easy matter to verify clinically the effect of medicines. But here, as in provings, Hahnemann was a master, and his verifications of provings were, indeed, not cures. The effect of remedies according to his *Organon*, should be prompt, pleasant, and permanent. No dangerous aggravations, and no trouble—some sequelæ should occur in homœopathic practice. In acute cases the stages of the disease should be shortened, and relief of the violent and dangerous symptoms should soon set in. And in chronic cases not only a palliation takes place, but complete cures are the rule, if we only mind Hahnemann's advice: "Macht's nach, aber macht's genau nach."

